# Supplementary material for: A high-resolution mRNA expression time course of embryonic development in zebrafish
Source: eLife. 2017 Nov 16;6:e30860. doi: 10.7554/eLife.30860 (PMC5690287; doi:10.7554/eLife.30860)
Supplement: Supplementary file 6. [file elife-30860-supp6.zip › biolayout-clusters-files/Cluster224-genes.html]

Cluster224


# Cluster224: Genes

| Ensembl ID | Gene Name | Chr | Start | End | Biotype |
| --- | --- | --- | --- | --- | --- |
| ENSDARG00000038566 | gnptg | 24 | 37450449 | 37460138 | protein\_coding |
| ENSDARG00000056892 | mpp6a | 16 | 21636543 | 21683743 | protein\_coding |
| ENSDARG00000018296 | rev1 | 9 | 21982696 | 22006721 | protein\_coding |
| ENSDARG00000023958 | rnf181 | 5 | 26155868 | 26165035 | protein\_coding |
| ENSDARG00000044203 | sumf1 | 6 | 39916317 | 39922569 | protein\_coding |
| ENSDARG00000078935 | ttll4 | 9 | 14141272 | 14172167 | protein\_coding |
